# A descriptive evaluation of a job analysis survey in the chiropractic profession in Switzerland – an update after more than 10 years

**DOI:** 10.1186/s12998-024-00544-1

**Published:** 2024-06-24

**Authors:** Mirjam Baechler, Nina Yerly, Lucas Tauschek, Petra Schweinhardt, Brigitte Wirth

**Affiliations:** Integrative Spinal Research Group, Department of Chiropractic Medicine, Balgrist University Hospital and University of Zurich, Forchstr. 340, Zurich, 8008 Switzerland

**Keywords:** Chiropractic, Female, Imaging, Interprofessional, Job satisfaction, Questionnaire, Survey

## Abstract

**Objectives:**

The aim of this cross-sectional study was to update the results of the first Swiss Job Analysis Survey in 2009 with regard to the demographics of the chiropractors and their patients, practice characteristics, interprofessional collaboration, the importance of imaging, and job satisfaction.

**Methods:**

In April 2020, the adapted 2009 questionnaire was electronically sent to all members of the Swiss Chiropractic Association ChiroSuisse (*N* = 316). Only complete questionnaires were included in the descriptive analysis. Demographic data were compared to all ChiroSuisse members.

**Results:**

The response rate was 76.3%. The mean age of the participants was 49.9 ± 12.3 years and 62.2% were male. Among the younger chiropractors (≤ 15 years of professional experience), 51.6% were male. Almost half of the participants worked in a joint office and one in five worked in a multidisciplinary setting. The typical chiropractic patient was middle-aged, female and suffered most frequently from acute lower back/pelvis pain and second most frequently from neck pain. Diversified osseous adjustment was the most commonly used technique, followed by advice on activities of daily living and trigger point therapy. Images (X-ray, CT, MRI) were ordered in less than 20% of the patients. 95% of the chiropractors were satisfied with their career choice.

**Conclusions:**

No changes to 2009 were observed in terms of the typical patient or the applied techniques. However, the Swiss chiropractors were more experienced, to a larger proportion female, more often worked in multidisciplinary settings, and ordered fewer images. Job satisfaction among Swiss chiropractors was high.

**Supplementary Information:**

The online version contains supplementary material available at 10.1186/s12998-024-00544-1.

## Background

In 2007, chiropractic became one of five recognized university medical professions in Switzerland. One year later, the first chair of Chiropractic Medicine in Switzerland was established at the Faculty of Medicine at the University of Zurich (UZH) and the 6-year direct entry undergraduate chiropractic program was started [1]. Since 2008, chiropractic students at the UZH complete a Bachelor of Medicine (focus Chiropractic Medicine) and a Master in Chiropractic Medicine [1]. This is followed by a mandatory fully accredited 2.5 years postgraduate program (‘assistancy’) that leads to the authorization to work independently [2, 3]. The assistancy entails working under the supervision of an experienced chiropractor (principal), a 4-month full time hospital rotation working in an integrated setting with relevant medical specialties (mandatory since the early 2000’s) and structured educational modules at the Swiss Academy of Chiropractic. The chiropractic profession has attained a high level of integration into mainstream healthcare in Switzerland. Chiropractors are first contact providers in the primary care setting with imaging rights including owning and operating X-ray machines as well as ordering advanced imaging, limited prescription rights, and the right to directly refer to specialists.

The first Swiss Job Analysis Survey, was conducted in 2009 [1]. The main findings were that the job profile of chiropractic in Switzerland is comparable with other countries in terms of the conditions treated (most frequently lower back pain, second most frequently neck pain), the patient age groups seen (primarily middle age) and the treatment methods used (primarily diversified osseous adjustment, followed by advice on activities of daily living, soft tissue techniques, and therapeutic exercises). However, the survey also pointed to much better interprofessional relationships, demonstrated by the referral patterns to and from chiropractors, compared to other countries [1]. Since then, in 2017, Beliveau and colleagues provided a global overview of the characteristics of chiropractic services and the patients seeking care and reported similar results [4].

Changes in healthcare delivery, i.e. in practice characteristics, are occurring gradually over time [5], but the time interval of more than ten years since the 2009 Swiss Job Analysis Survey might be long enough to capture some potential changes: (i) the first graduates of the integrated Swiss program have entered professional life, which might have, given the small number of chiropractors in Switzerland, an impact on practice characteristics; (ii) there has been a strive for an increase in interprofessional collaboration and education at all levels in Switzerland [6]; (iii) imaging guidelines have been published [7] and initiatives on decreasing the use of routine imaging in low back pain were rolled out [8]; (iv) as in many other countries, more women than men are enrolled in the medical and chiropractic programs in Switzerland [9, 10], which will most likely lead to a shift in the male to female ratio of chiropractors. A secondary analysis of the 2009 Swiss Job Analysis Survey showed that females were working fewer hours and seeing fewer patients per week. The authors speculated that this might lead to a shortage of chiropractors. However, whether there is a clear link between an increase in females in medical professions and workforce shortages or whether this is due to more complex social changes is the subject of an ongoing debate [11]. Lastly, in the 2009 Swiss Job Analysis Survey, no questions were asked about job satisfaction which was deemed an important gap because it seems highly relevant to the recruitment of future students as well as the retention of chiropractors in the workforce.

Thus, the aim of the present study was to update the results of the 2009 Swiss Job Analysis Survey with regard to the demographics of the chiropractors and their patients as well as the practice characteristics, and to place an additional focus on current topics such as male to female ratio, interprofessional collaboration, the importance of imaging, and job satisfaction.

## Methods

### Study design

With the authorization of the authors of the 2009 Swiss Job Analysis Survey, this cross-sectional study used a questionnaire that was built on the previous, but has been slightly adapted with respect to literature published since then in the fields of chiropractic [4, 10, 12–19] and osteopathy [20, 21]. The study team composing the questionnaire consisted of delegates of ChiroSuisse, which is the professional association for chiropractors in Switzerland, as well as members of the research team of the Department of Chiropractic Medicine at the UZH, physically located at the Balgrist University Hospital in Zurich, Switzerland. As a new topic, the questions on job satisfaction from a Norwegian job survey [17] were added. The respective questions were provided by the original authors and translated into English by a Norwegian speaking research team member. The questionnaire language was English. The final questionnaire (Additional file 1) was implemented in the software REDCap (version 8.3.2), which is a secure web-based application designed to support data capture in research [22], and was pilot tested by study team members. The Ethics Committee of the canton of Zurich confirmed that the research project was not subject to the Human Research Act (HRA) and that authorization by the committee was not required (BASEC-Nr Req-2020-00140).

### Study protocol

In April 2020, after informing about the planned survey at the General assembly of ChiroSuisse in September 2019, a survey invitation was sent by email to all fully licensed chiropractors (*N* = 287) and all chiropractors undergoing postgraduate training (*N* = 29) registered with ChiroSuisse, which represents approximately 98% of all chiropractors in Switzerland by estimation (personal communication with the ChiroSuisse president). This email included an individual link, the information that all answers would remain anonymous and an estimated time requirement of 30 min. Because of the COVID-19 pandemic, a video explaining the importance of the survey was shown three weeks later at the online General Assembly of ChiroSuisse and it was communicated that continuing education (CE) credits were given for fully completed questionnaires to increase the response rate. Reminders were sent every other week until the end of June 2020, and data was acquired between April 14 and July 8, 2020. A member of the research team of the Department of Chiropractic Medicine at Balgrist University Hospital acted as database administrator, could be contacted for questions and guaranteed data anonymity as only he had access to the original database.

### Data analysis

Like in the 2009 Swiss Job Analysis Survey [1], survey data was analyzed descriptively: for categorical data, the percentage of chiropractors who chose this category was given for each category (proportions) and for continuous variables, mean and standard deviation were calculated. To quantify the representativeness of the results, the demographic data of the participating chiropractors including age and sex were compared with the demographic data of all ChiroSuisse members. Continuous data was compared using unpaired, two-sided Student’s t-tests, and categorial data using Pearson chi-squared tests. The level of significance was set to 0.05 and IBM SPSS Statistics for Windows (Version 25.0. Armonk, NY: IBM Corp.) was used.

## Results

### Response rate and representativeness of the results

The response rate was 76.3% with 241 out of 316 ChiroSuisse members fully completing the survey. In addition, six participants filled in the survey only partially (four partly completed the first part on demographics, one fully completed the demographic part and another participant only completed the first two parts on demographics and practice characteristics). Due to the very limited information provided, these six cases were excluded and the 241 complete cases were analyzed. There was no statistically significant difference between the participants and all the ChiroSuisse members in terms of age (*t*_(555)_=-0.48, *p* = 0.631), and sex (χ^2^_(1)_ = 2.0, *p* = 0.157).

### Demographics of responding chiropractors

Characteristics of the responding chiropractors are summarized in Table 1: 62.2% were male, mean age was 49.9 ± 12.3 years (standard deviation; SD) (range 26–80 years), 25% were close to retirement (56–65 years) and 10.8% were older than the Swiss retirement age of 65 years. 66.5% of the participating chiropractors had more than 15 years of professional experience, with 34.4% having even more than 25 years. Among the younger chiropractors with ≤ 15 years of professional experience, 51.6% were male, and of the 23 chiropractors (9.6%) who graduated at the University of Zürich in Switzerland, 26.1% were male. Of all participants, 35.0% were involved in education, mostly as principals (17.0%), teachers for postgraduate students (12.0%), or clinical supervisors (10.4%) (for details see Additional file 2, Table S1). The continuing education of the participants consisted of 68.3% attending courses and seminars offered by the Swiss Chiropractic Academy and of 67.1% attending seminars and conferences in the chiropractic and multidisciplinary settings (for details see Additional file 2, Table S2). The most common institution that chiropractors graduated from was the Canadian Memorial Chiropractic College, followed by the University of Western States (for details see Additional file 2, Table S3).

**Table 1:** Demographics of responding chiropractors working in Switzerland

Age (years ± standard deviation)	49.9 ± 12.3
Gender (m/f/other) (%)	62.2/37.8/0.0
Linguistic region of the practice^1^	
German-speaking (%)	68.9
French-speaking (%)	29.9
Italian-speaking (%)	4.6
Rhaeto-Romanic-speaking (%)	1.2
Postgraduate trainee (assistantship) (yes/no) (%)	7.9/92.1
Professional experience since completing assistantship	
< 2 years (%)	5.4
2–4 years (%)	6.3
5–15 years (%)	21.7
16–25 years (%)	32.1
> 25 years (%)	34.4
Hospital rotation completed (yes/no) (%)	45.8/54.2
Involved in education (yes/no) (%)	35.0/65.0

^1^ Total not 100% because more than one linguistic region could be marked

### Practice characteristics

Practice characteristics are summarized in Table 2: 31.8% of the participating chiropractors worked more, the rest less than 40 h per week. They mostly spent between 31 and 45 min with their patients at the first visit and between 11 and 15 min at subsequent visits. As for office organization, 37.8% of the participants were individual practitioners, 46.9% worked in a joint office with two or more chiropractors and 21.2% worked in a multidisciplinary setting. In addition, 10.8% indicated to have staff privileges to treat patients in a hospital.

**Table 2:** Practice characteristics of responding chiropractors in Switzerland

Hours per week you practice	% of chiropractors
< 11 h	2.5
11–20 h	5.8
21–30 h	22.1
31–40 h	37.9
41–50 h	23.8
51–60 h	4.2
> 60 h	3.8
Number of patients you personally treat per week	
< 50	7.9
50–99	41.0
100–149	30.5
150–199	10.0
200–249	5.4
250–300	2.9
> 300	2.1
Number of *NEW* patients you personally treat per week	
0	0.8
1–3	9.5
4–6	31.1
7–9	27.8
10–12	17.4
16–20	2.5
> 20	2.5
How much time do you spend on average at first visit	
11–15 min	2.5
16–30 min	24.5
31–45 min	61.0
46–60 min	11.6
61–75 min	0.4
How much time do you spend on average at subsequent visit	
0–5 min	0.8
6–10 min	25.7
11–15 min	51.5
16–30 min	21.6
31–45 min	0.4
How soon can a new patient get an *URGENT* appointment with you?	
Same day	49.4
1–2 days	36.9
3–4 days	6.2
5–7 days > 1 week	2.9 4.6
How soon can a new patient get a *NON-URGENT* appointment with you?	
Same day	3.7
1–2 days	28.6
3–4 days	31.1
5–7 days > 1 week	17.4 19.1
Office organization (“Which description best characterizes your role in the office where you work? Mark all that apply.“)	
Individual practitioner/only chiropractor in office	37.8
One of two or more chiropractors in office	46.9
Practitioner in multidisciplinary office	21.2
Other	2.9
Do you practice in more than one office location? (yes/no)	18.7/81.3
Do you have staff privileges to treat patients in a hospital? (yes/no)	10.8/89.2

### Mode of operation

#### Treatment modalities

The most commonly used technique by the participating chiropractors was diversified osseous adjustments, which was applied in 72.1% of the treated patients, followed by advice on activities of daily living, trigger point therapy, therapeutic exercises and lifestyle counselling (Table 3). Most of the chiropractors reassessed their patients between the first and fifth visit (for details see Additional file 2, Table S4).

**Table 3:** Treatment modalities most commonly used by responding chiropractors in Switzerland

Treatment modality (“With what percentage of your patients do you use the following techniques/treatments?“)	% of patients (% ± SD)
Diversified osseous adjusting	72.1 ± 27.2
Activities of daily living advice	53.4 ± 30.7
Trigger point therapy	47.2 ± 29.9
Therapeutic exercises	40.5 ± 30.2
Lifestyle counselling	38.3 ± 31.9
Physical therapy modalities (ultrasound, electrotherapy, heat/cold, traction, etc.)	34.4 ± 30.6
Mobilization	31.9 ± 29.6
Rehabilitation techniques	24.0 ± 26.0
Drop technique	22.4 ± 26.6
Massage	19.6 ± 29.6
Cox flexion distraction	18.9 ± 21.8
Active muscle release technique (Graston etc.)	18.7 ± 23.8
Nutritional counselling	15.4 ± 20.1
Pain medication prescription	14.1 ± 15.9
Gonstead osseous adjusting	12.0 ± 25.4
Applied kinesiology	11.5 ± 25.1
Other	10.9 ± 20.6
Strapping/taping	10.8 ± 13.5
Sacrooccipital technique	10.5 ± 19.9
Dry needling	9.6 ± 16.7
Activator technique	8.1 ± 13.8
Orthotic prescription	7.4 ± 10.3
Trigger point injections	6.4 ± 15.9
Network technique	3.9 ± 15.9
Acupuncture	2.5 ± 7.6

SD=standard deviation

#### Imaging

X-rays were more often ordered or taken than were CT or MR imaging, but most of the chiropractors prescribed X-ray and CT/MR imaging for fewer than 20% of their patients. Almost half of the chiropractors took the X-rays in their own practice, and 39.0% had them taken in an imaging center or hospital. X-rays were mostly taken and interpreted by the chiropractors themselves, even when a radiologist’s report was available. The CT and MR images were interpreted based on the radiologists’ reports, but own conclusions were always drawn (Table 4).

**Table 4:** Frequency and interpretation of diagnostic imaging in responding chiropractic practices in Switzerland

X-ray prescription *(“For what percentage of your patients do you order or take an x-ray?“)*	% of chiropractors
0% of patients	0.4
1–20% of patients	74.6
21–40% of patients	14.6
41–60% of patients	5.4
61–80% of patients	4.2
81–100% of patients	0.8
X-ray interpretation *(“Who interprets the x-rays of your patients?“)*	
I generally interpret all of the images of my patient, even if I have a radiologist’s report.	63.9
The interpretation is generally done by a radiologist, but I always draw my own conclusions in addition to the radiologist’s report.	26.6
I only interpret the images that I take in my practice and rely on the report for images taken elsewhere.	8.7
I do not interpret any of the images of my patients, relying solely on a radiologist’s report.	0.8
Taking of X-rays *(“Do you primarily delegate taking X-rays to non-chiropractic member of your staff?“)*	
Yes	19.5
No	45.6
I do not take X-rays in my practice	34.9
Place where X-rays are taken *(“When radiographs are indicated for your patients where are they done?“)*	
Nearly all are taken in my practice At an imaging center or hospital Some are taken at my practice and others are referred to another facility	46.5 39.0 14.5
CT or MRI imaging prescription (“*For what percentage of your patients do you order CT or MRI imaging?“)*	
0% of patients	0.0
1–20% of patients	94.6
21–40% of patients	4.6
41–60% of patients	0.0
61–80% of patients	0.8
81–100% of patients	0.0
CT or MRI imaging interpretation *(“Who interprets the x-rays of your patients?“)*	
I generally interpret all of the images of my patients, even if I have a radiologist’s report.	23.7
The interpretation is generally done by a radiologist, but I always draw my own conclusion in addition to the radiologist’s report.	68.5
I do not interpret any of the images of my patients, relying solely on a radiologist’s report.	7.9

#### Referrals

New patients were most frequently referred by patients, followed by medical practitioners (for details see Additional file 2, Table S5). Among the latter, as summarized in Table 5, patients were mostly referred to chiropractic care by family practitioners, followed by internists, orthopedic surgeons, gynecologists and physiotherapists. Vice versa, chiropractors mostly referred patients to physiotherapists, family practitioners, massage therapists, orthopedic surgeons and internists (for details see the Additional file 2, Table S6). Despite these collaborations, 90% of the chiropractors indicated that the collaboration between different health professionals could be improved, while 5% were satisfied with the current state of collaboration and 5% were uncertain.

**Table 5:** Referrals from other health professionals to chiropractic care and referrals by chiropractors to other health professionals in responding chiropractic practices in Switzerland

	Often (1-2x/week) (% of chiropractors)	Routinely (> 2x/week) (% of chiropractors)
Referrals from other health professionals to chiropractic care *(“How frequently have the following health care professionals made referrals to you during the past year?“)*		
Family practitioner	33.6	38.2
Internist	19.5	5.0
Orthopedic surgeon	13.3	4.1
Gynecologist	14.1	2.1
Physiotherapist	10.4	1.2
Referrals by chiropractors to other health professionals *(“How frequently have you made referrals to the following health professionals during the past year?“)*		
Physiotherapist	25.7	10.4
Family practitioner	27.8	7.1
Massage therapist	12.9	3.7
Orthopedic surgeon	10.4	2.1
Internist	9.1	2.1

#### Job satisfaction

Job satisfaction among Swiss chiropractors was strikingly high: almost all were satisfied with their career choice, experienced sufficient daily professional challenges, and felt that their income appropriately reflected the time and effort they put into their work (Table 6).

**Table 6:** Job satisfaction of responding chiropractors in Switzerland

Are you generally satisfied with your career choice as a chiropractor?	% of chiropractors
Yes	95.0
No	0.8
Uncertain	4.2
Do you experience sufficient daily professional challenges?	
Yes	91.7
No	2.5
Uncertain	5.8
Does your income appropriately reflect the time and effort you put into work?	
Yes	74.2
No	16.7
Uncertain	9.2

### Patient characteristics

Chiropractic patients seem to be mostly female and middle-aged: 58.3% of the chiropractors indicated treating 51–75% female patients, 66.8% indicated that 26–50% of their patients were between 31 and 50 years and 61.4% reported that 26–50% of their patients were between 51 and 64 years old. Young adults between 18 and 30 years of age and people between 65 and 74 years of age made up to 26–50% of the patients for 23.2% and 29.0% of the chiropractors, respectively, while children and people aged ≥ 75 years were treated relatively seldomly and made up 1–25% of the patients in most practices (for details see the Additional file 2 Table S7).

The treated patients mostly suffered from acute symptoms of less than four weeks duration: 23.2% of the responding chiropractors treated at least 50% of patients with acute symptoms, while chronic patients (symptom duration > 12 weeks) made up to 1–25% of the patients in most practices, and only a minority of the responding chiropractors treated mostly chronic patients (for details see the Additional file 2, Table S8).

The patients’ most common complaint was lower back/ pelvis pain without leg pain: more than 11.2% of the chiropractors estimated that more than half of the treated patients suffered from lower back or pelvis pain without leg pain, while the treatment of patients suffering from lower back/pelvis pain with leg pain was slightly less common with 6.1%. Neck pain was the second most commonly treated complaint: 3.7 of the chiropractors estimated that more than 50% of their patients suffered from neck pain without arm pain, 3.3% from headache with neck pain and 1.6% of neck pain with arm pain. With 2.9% of chiropractors estimating that more than 50% of their patients suffered from midback pain, the latter was also a commonly treated complaint (for details, see Additional file 2, Table S9).

## Discussion

The main findings of this study were that the characteristics of the typical chiropractic patient and the most commonly applied chiropractic techniques have not changed since the 2009 Swiss Job Analysis Survey apart from the fact that fewer images were ordered. The chiropractic profession in Switzerland has become more female, is increasingly embedded in multidisciplinary settings and the chiropractors are highly satisfied with their career choice.

The response rate is slightly higher than that of the Swiss Job Analysis Survey from 2009 (76% vs. 70%) and comparable to that of studies from other countries [13, 17, 18, 23, 24]. The structured reminders for participation and being able to obtain 5 CE credits might have contributed to this high response rate [25].

The job profile of chiropractic in Switzerland is comparable to the 2009 Swiss Job Analysis Survey in terms of the main conditions treated (lower back/pelvis pain, neck pain, headache, midback pain in decreasing order) and the main age groups and gender of the patients, i.e. primarily middle aged and female. This finding is also in line with the overall international literature as described by Beliveau and colleagues in a scoping review from 2017 and other recent international literature [16, 17, 26]. However, there were shifts at both ends of the patient age spectrum in the present survey compared to those in the 2009 survey: fewer chiropractors reported seeing patients under 5 years of age and more chiropractors reported seeing patients older than 65 years with 87% reporting that 1–25% of their patients were 85 years of age and older. While the latter is most likely explained by the typical aging demographic in a Western country, the first cannot be fully explained at this time. The 2020 survey also included a third gender category of ‘other’ which was not further specified. One in six chiropractors reported seeing between 1 and 25% of patients in this category, which indicates that they not only recognize more than two gender categories but are also able to provide for an open environment in which the patients feel comfortable revealing their gender [27]. The main treatment methods applied are still the same as in the 2009 Swiss Job Analysis Survey [1] and compare well to the international literature [4]: diversified osseous adjustments, advice on activities of daily living, trigger point techniques and therapeutic exercise are the mainstays of Swiss chiropractors and these seem to represent a typical package of care.

The chiropractic profession in Switzerland is slowly growing with a total population of 287 fully licensed members in 2020 (vs. 260 in 2009). The percentage of female chiropractors increased from 29 to 38%, but the profession is still male dominated, as in some other countries (Norway [17], Australia [12]), while others have more equal gender distributions (Canada [28], South Africa [16]) or is female dominated (Denmark [18]). The proportion of chiropractors with > 25 or 16–25 years of practice experience have each increased to 32% (vs. 18% and 26% in 2009). For the development of the chiropractic profession in Switzerland, it is important to note that 11% of the chiropractors were above and 25% were close to the retirement age, and that of those chiropractors with less than 15 years of experience 48% were female and of those who graduated in Switzerland (*N* = 23), 74% were female. The observed shifts in graduating institutions are most likely driven by the start of the publicly funded chiropractic education in Switzerland in 2008.

Along with the shift in the ratio of female to male chiropractors and a more experienced chiropractic profession, there have been remarkable changes in practice characteristics over the past ten years, although changes in healthcare practices, at least in the case of de-implementation of non-evidence based techniques, have been shown to take several decades [5]. For example, fewer chiropractors work in an individual office (38% vs. 48% in 2009) and there is a trend toward fewer hours spent in practice as more chiropractors reported working 40 h or less (68% vs. 57% in 2009). There also seems to be a trend toward more time spent with patients during subsequent visits: fewer chiropractors reported spending 6–10 min (26% vs. 29% in 2009), the majority still spent 11–15 min (51% vs. 58% in 2009) and more chiropractors spent 16–30 min (22% vs. 11% in 2009). This might be due to increased demands on documentation, a more complex patient case mix or simply a change in practice behavior. All of these factors lead to fewer patients being seen overall. This development is also accompanied by longer waiting times for urgent and non-urgent appointments for new patients: The proportion of chiropractors reporting that an urgent appointment on the same day was possible decreased to 49% (vs. 61% in 2009). A non-urgent same day appointment was possible only for 4% of the chiropractors (vs. 13% in 2009) and 19% (vs. 10%) reported waiting times > 1 week for non-urgent patients. It ought to be easier to maintain a part time practice when sharing office space so some of the factors above might be linked. Additionally, since the Chair of Chiropractic Medicine was established and the undergraduate program at the UZH was started in 2008 [1] more diverse professional pathways have become available for chiropractors because of the demand for educators and researchers, which in turn might have contributed to fewer hours spent in practice.

A large proportion of the profession is close to or above retirement age and is expected to retire in the next few years. This development and future possible scenarios for the chiropractic profession have also been shown in a recent needs analysis commissioned by ChiroSuisse [29]. It is foreseeable that the profession will become even more female in the future as the current student body at the UZH [9] and most likely internationally at chiropractic institutions is more female than male. Whether this is the only factor contributing to an ongoing trend toward fewer hours in practice needs to be better understood. Practice patterns, especially work hours and the number of patients seen, might also be dynamic throughout a chiropractor’s professional life due to changes in work life balance, changing care needs of family members (e.g., children, elderly parents) and potentially physical restrictions as chiropractors age.

One particular area of interest was the development of interprofessional relationships and collaboration by the chiropractic profession in Switzerland because this had been identified in the previous survey to be at a more advanced level than in many other countries. The results show a continuing trend in this as evidenced by a number of factors. The percentages of chiropractors working in multidisciplinary offices and who have staff privileges to treat patients at a hospital have almost doubled to 21% and 11%, respectively. The latter might also be related to the many chiropractors contributing to chiropractic education through the role of part-time clinical supervisors for the teaching clinics which are situated in university hospitals in Zurich and Lausanne and opened in 2014 and 2020, respectively. Overall, chiropractors reported that one-third of their new patients were referred by other health professionals. The referral patterns to and from chiropractors have not changed much regarding the professions that are mentioned the most often. However, specialists such as orthopedic surgeons, neurologists and neurosurgeons seem to refer more often to chiropractors than in 2009. Interestingly, despite this continuing trend in interprofessional collaboration, the vast majority of chiropractors (90%) stated that interprofessional collaboration could improve. Almost half of the chiropractors (46%) stated that they had completed a hospital rotation. This seems to go back to before the hospital rotation was made a mandatory part of the postgraduate program in the early 2000s. Hence, a large number of chiropractors in Switzerland have been exposed to interprofessional work at some time during their educational pathway or professional life. The questions regarding how continuing education credits were obtained were changed to include a differentiation between the chiropractic field and multidisciplinary setting with a large proportion of chiropractors stating that they obtain it in a mixed setting. Overall, interprofessional collaboration is likely driven by factors at the systems and individual levels. At the systems level, legal recognition and reimbursement schemes are most likely to be easier for chiropractors in Switzerland. It seems plausible that healthcare professionals would be more likely to refer to another recognized healthcare professional whose service is also covered by the same reimbursement schemes. At the level of the individual chiropractor, having such a large proportion of chiropractors who at some point worked in an integrated setting and participated in continuing education with other healthcare professionals certainly influences the further integration of the chiropractic profession.

A rather unexpected finding concerns the use of imaging by the chiropractic profession in Switzerland. There was a marked shift with 75% of the chiropractors reporting using X-ray imaging for only up to 20% of their patients (vs. 40% in the 2009 survey) and only 1% of chiropractors stating that they would use X-ray imaging for more than 80% of their patients (vs. 4% in 2009). Interestingly, fewer normal X-ray prescriptions were not replaced by an increased use of advanced imaging. The rate of advanced imaging showed a similar trend albeit at a lower level with 95% reporting using it for up to 20% of their patients (vs. 77% in 2009) and 5% reporting using it for up to 40% of their patients (vs. 19% in 2009). This shift might be related to fewer chiropractors owning their own X-ray machine as expressed by the decrease in the number of chiropractors stating that they take nearly all the X-rays at their office (47% vs. 55% in 2009). Alternatively, it could be a sign of better adherence to imaging [7] and practice guidelines [30, 31] or of responsiveness to more general healthcare initiatives such as ‘smarter medicine’, the Swiss version of Choosing Wisely, that clearly advocate not using routine X-rays for uncomplicated low back pain in the first six weeks [32]. Unfortunately, the question on how many patients already had X-rays or advanced imaging at the time of their chiropractic intake was not asked in either of the surveys, but would have complemented the understanding of this important topic.

Finally, 95% of the chiropractors were satisfied with their career choices, 92% experienced sufficient professional challenges and 74% stated that their income was reflective of the time and effort put into work, which is in line with international findings [17]. These results can be used for the recruitment of future students and might be a good explanation for the very low percentage of chiropractors leaving the profession.

### Limitations of the survey

As with any self-report survey, the results are only as valid as the honesty of the participants’ answers. Since the survey was administered anonymously, no attempt was made to validate any of the results. Of all chiropractors registered at ChiroSuisse, 24% did not respond to this survey. However, as the participants were representative of the whole chiropractic profession in Switzerland with respect to age and sex, one can assume that the results of the present study are generalizable to all Swiss chiropractors. The survey was conducted during the COVID-19 pandemic in Switzerland. Last-minute adaptations to the questions with prompts to refer to pre-COVID times had to be put in place and might have influenced the answers of individual chiropractors. The timing of the questionnaire could also have positively influenced the response rate as it was difficult to obtain CE credits during this time and due to the restrictions to only treat acute patients in the practices, the chiropractors might have had more time to participate.

## Conclusions

This update of the 2009 Swiss Job Analysis Survey showed no changes in terms of the typical chiropractic patient or the techniques applied by the chiropractors. However, relevant shifts are seen in the chiropractic profession itself, which is more female and more experienced than in the previous survey. This is accompanied by a shift away from individual chiropractor offices toward multi-chiropractor and/or multi-disciplinary offices, which shows that the high level of interprofessional collaboration already observed in 2009 continues. A surprising finding was the large decrease in the prescription of imaging. The participating chiropractors were highly satisfied with their career choice, which is promising for the future of the chiropractic profession in Switzerland.

### Electronic supplementary material

Below is the link to the electronic supplementary material.

Additional file 1: Survey

Additional file 2: Supplementary results

## Data Availability

The datasets used and/or analysed during the current study are available from the corresponding author upon reasonable request.

## Abbreviations

ADL: Activities of daily living
HRA: Human Research Act
LBP: Low back pain
UZH: University of Zurich
