# Supplementary material for: A descriptive evaluation of a job analysis survey in the chiropractic profession in Switzerland – an update after more than 10 years
Source: Chiropr Man Therap. 2024 Jun 24;32:25. doi: 10.1186/s12998-024-00544-1 (PMC11197174; doi:10.1186/s12998-024-00544-1)
Supplement: Supplementary file 1 — Additional file 1: Survey [file 12998_2024_544_MOESM1_ESM.docx]

## Additional file 1: Survey

Questions are displayed in the same order as they appear in the text.

### Demographics of chiropractors

1. Gender

- Male
- Female
- Other

1. Year of birth (e.g. 1971)
2. Linguistic region of the clinic. (Mark all that apply.)

- German-speaking part of Switzerland
- French-speaking part of Switzerland
- Italian-speaking part of Switzerland
- Rhaeto-Romanic-speaking part of Switzerland

1. Are you currently an assistant?

- Yes, 1st year
- Yes, 2nd year
- Yes, 3rd year
- Yes, 4th year
- Yes, 5th year
- No

1. Years in practice since completing Assistantship

- Less than 2 years
- 2–4 years
- 5–15 years
- 16–25 years
- More than 25 years

1. Did you complete a hospital rotation during your assistantship?

- Yes
- No

1. Are you involved in education?

- Yes
- No

1. How are you involved in education? (Mark all that apply.)

- As a principal
- As a teacher for undergraduate students (bachelor and master level)
- As a teacher for postgraduate students (e.g., academy)
- As a teacher for continuing education (e.g., annual CE convention, multidisciplinary courses)
- As an examiner/as a member of the examining commission
- As a clinical supervisor
- Other (please specify

1. In which of the following continuing education opportunities do you participate? (Mark all that apply.)

| CE opportunities | None | Chiropractic field | Multidisciplinary setting | Both |
| --- | --- | --- | --- | --- |
| Reading journals | ☐ | ☐ | ☐ | ☐ |
| Attending conferences/seminars | ☐ | ☐ | ☐ | ☐ |
| Attending diploma courses | ☐ | ☐ | ☐ | ☐ |
| Attending hospital staff CE meetings | ☐ | ☐ | ☐ | ☐ |
| Online credit courses | ☐ | ☐ | ☐ | ☐ |
| Courses/seminars offered by Swiss  Chiropractic Academy | ☐ | ☐ | ☐ | ☐ |
| Other, please list: __________ | ☐ | ☐ | ☐ | ☐ |

1. Institution that conferred your chiropractic degree
   - AECC University College / Anglo-European College of Chiropractic
   - Canadian Memorial Chiropractic College
   - Cleveland Chiropractic College
   - Durban University of Technology
   - Institut France-Européen de Chiropratique, Ivry-Sur-Seine
   - Institut France-Européen de Chiropratique, Toulouse
   - Logan Chiropractic College
   - Los Angeles College of Chiropractic / Southern California University of Health Sciences
   - Macquarie University
   - Murdoch University
   - National College of Chiropractic / National University of Health Sciences
   - New York Chiropractic College
   - Northwestern Health Sciences University / Northwestern College of Chiropractic
   - Palmer College of Chiropractic
   - Palmer College of Chiropractic West
   - Texas Chiropractic College
   - Université du Québec à Trois-Rivières
   - University of Johannesburg
   - University of Southern Denmark
   - University of Western States, Chiropractic College
   - University of Zurich
   - Welsh Institute of Chiropractic, University of Glamorgan
   - Other (Please specify)

### Practice characteristics

1. Hours per week you practice

- Less than 11
- 11–20
- 21–30
- 31–40
- 41–50
- 51–60
- More than 60

1. Number of patients you personally treat per week

- Less than 50
- 50–99
- 100–149
- 150–199
- 200–249
- 250–300
- More than 300

1. Number of NEW patients you personally see per week

- 0
- 1–3
- 4–6
- 7–9
- 10–12
- 13–15
- 16–20
- More than 20

1. How soon can a NEW patient get an URGENT appointment with you?

- Same day
- 1–2 days
- 3–4 days
- 5–7 days
- More than 1 week

1. How soon can a NEW patient get a NON-URGENT appointment with you?

- Same day
- 1–2 days
- 3–4 days
- 5–7 days
- More than 1 week

1. How much time, on average, do you spend with a NEW patient on the first visit?

- 0–5 minutes
- 6–10 minutes
- 11–15 minutes
- 16–30 minutes
- 31–45 minutes
- 46–60 minutes
- 61–75 minutes
- 76–90 minutes
- >90 minutes

1. How much time, on average, do you spend with a patient on SUBSEQUENT visits?

- 0–5 minutes
- 6–10 minutes
- 11–15 minutes
- 16–30 minutes
- 31–45 minutes
- 46–60 minutes
- 61–75 minutes
- 76–90 minutes
- >90 minutes

1. Which description best characterizes your role in the office where you work? (Mark all that apply.)

- Individual practitioner/only chiropractor in office
- One of two or more chiropractors in office
- Practitioner in multidisciplinary office
- Other

1. Do you practice in more than one office location?

- Yes
- No

1. Do you have staff privileges to treat patients in a hospital?

- Yes
- No

1. Approximately what percentage of your time is spent on each of the following functions during a typical week? (Total should be approximately 100%.)

|  | None | 1–25% | 26–50% | 51–75% | 76–100% |
| --- | --- | --- | --- | --- | --- |
| Direct patient care | ☐ | ☐ | ☐ | ☐ | ☐ |
| Documentation of care | ☐ | ☐ | ☐ | ☐ | ☐ |
| Business management (personnel, marketing, etc) | ☐ | ☐ | ☐ | ☐ | ☐ |

### Mode of operation

1. With what percentage of your patients do you use the following techniques/treatments? (May add up to greater than 100% as patients may receive more than one therapy.)

Diversified osseous adjusting

Gonstead osseous adjusting

Drop technique

Activator technique

Applied kinesiology

Cox Flexion distraction

Network technique

Mobilization

Active muscle release technique (Graston etc)

Sacrooccipital technique

Physical therapy modalities (ultrasound, electrotherapy, heat/cold, traction, etc)

Massage

Trigger point therapy

Trigger point injections

Dry needling

Acupuncture

Rehabilitation techniques

Orthotic prescription

Therapeutic exercises

Nutritional counseling

Activities of daily living advice

Pain medication prescription

Strapping/taping

Lifestyle counseling

Other

1. At what visit interval do you reassess your patients for whom you are providing ongoing care?

- 1 visit
- 2–3 visits
- 4–5 visits
- 6–7 visits
- 8–9 visits
- After 10 or more visits
- I do not reassess

1. For what percentage of your patients do you order or take an x-ray?

- 0%
- 1–20%
- 21–40%
- 41–60%
- 61–80%
- 81–100%

1. When radiographs are indicated for your patients where are they done?

- Nearly all are taken in my practice
- At an imaging center or hospital
- Some are taken at my practice and others are referred to another facility

1. Do you primarily delegate taking x-rays to non-chiropractic member of your staff?

- Yes
- No
- I do not take x-rays in my office

1. Who interprets the x-rays of your patients?

- I generally interpret all of the images of my patients, even if I have a radiologist’s report.
- I only interpret the images that I take in my practice and rely on the report for images taken elsewhere.
- I do not interpret any of the images of my patients, relying solely on a radiologist’s report.
- The interpretation is generally done by a radiologist, but I always draw my own conclusions in addition to the radiologist’s report.

1. For what percentage of your patients do you order CT or MRI imaging?

- 0%
- 1–20%
- 21–40%
- 41–60%
- 61–80%
- 81–100%

1. Who interprets the CT or MRI imaging of your patients?

- I generally interpret all of the images of my patients, even if I have a radiologist’s report.
- I do not interpret any of the images of my patients, relying solely on a radiologist’s report.
- The interpretation is generally done by a radiologist, but I always draw my own conclusions in addition to the radiologist’s report.

1. Please approximate the percentage of your new patients received from the following sources. (Total should be approximately 100%)

ChiroSuisse website

Your own personal website

Patient contact with ChiroSuisse

Sign/location of your practice

Word-of-mouth/patient referrals

Chiropractic patients association

Referral from medical practitioner

Referral from other chiropractor

Referral from other health care practitioner

Other

Don’t know

1. How frequently have the following health care professionals made REFERRALS TO YOU during the past year? (Rarely (<1/mon), sometimes (1-3/mon), often (1-2/wk), routinely (>2/wk)) Please look at the year before the COVID-19 crisis started in Switzerland.

| Practitioner | Never | Rarely (<1/mon) | Sometimes (1-3/mon) | Often (1-2/wk) | Routinely (>2/wk) |
| --- | --- | --- | --- | --- | --- |
| Acupuncturist | ☐ | ☐ | ☐ | ☐ | ☐ |
| Dentist | ☐ | ☐ | ☐ | ☐ | ☐ |
| Family practitioner | ☐ | ☐ | ☐ | ☐ | ☐ |
| Internist | ☐ | ☐ | ☐ | ☐ | ☐ |
| Massage therapist | ☐ | ☐ | ☐ | ☐ | ☐ |
| Neurologist | ☐ | ☐ | ☐ | ☐ | ☐ |
| Neurosurgeon | ☐ | ☐ | ☐ | ☐ | ☐ |
| Nutritionist | ☐ | ☐ | ☐ | ☐ | ☐ |
| OB/GYN | ☐ | ☐ | ☐ | ☐ | ☐ |
| Orthopedic surgeon | ☐ | ☐ | ☐ | ☐ | ☐ |
| Orthopedic technician | ☐ | ☐ | ☐ | ☐ | ☐ |
| Other chiropractor | ☐ | ☐ | ☐ | ☐ | ☐ |
| Pediatrician | ☐ | ☐ | ☐ | ☐ | ☐ |
| Physiatrist / Rheumatologist | ☐ | ☐ | ☐ | ☐ | ☐ |
| Physical Therapist | ☐ | ☐ | ☐ | ☐ | ☐ |
| Psychologist / Psychiatrist | ☐ | ☐ | ☐ | ☐ | ☐ |
| Surgeon | ☐ | ☐ | ☐ | ☐ | ☐ |
| Other | ☐ | ☐ | ☐ | ☐ | ☐ |

1. How frequently have YOU MADE REFERRALS TO the following health professionals during the past year? (Rarely (<1/mon), sometimes (1-3/mon), often (1-2/wk), routinely (>2/wk)) Please look at the year before the COVID-19 crisis started in Switzerland.

| Practitioner | Never | Rarely (<1/mon) | Sometimes  (1–3/mon) | Often  (1–2/wk) | Routinely (>2/wk) |
| --- | --- | --- | --- | --- | --- |
| Acupuncturist | ☐ | ☐ | ☐ | ☐ | ☐ |
| Dentist | ☐ | ☐ | ☐ | ☐ | ☐ |
| Family practitioner | ☐ | ☐ | ☐ | ☐ | ☐ |
| Internist | ☐ | ☐ | ☐ | ☐ | ☐ |
| Massage therapist | ☐ | ☐ | ☐ | ☐ | ☐ |
| Neurologist | ☐ | ☐ | ☐ | ☐ | ☐ |
| Neurosurgeon | ☐ | ☐ | ☐ | ☐ | ☐ |
| Nutritionist | ☐ | ☐ | ☐ | ☐ | ☐ |
| OB/GYN | ☐ | ☐ | ☐ | ☐ | ☐ |
| Orthopedic surgeon | ☐ | ☐ | ☐ | ☐ | ☐ |
| Orthopedic technician | ☐ | ☐ | ☐ | ☐ | ☐ |
| Other chiropractor | ☐ | ☐ | ☐ | ☐ | ☐ |
| Pediatrician | ☐ | ☐ | ☐ | ☐ | ☐ |
| Physiatrist / Rheumatologist | ☐ | ☐ | ☐ | ☐ | ☐ |
| Physical therapist | ☐ | ☐ | ☐ | ☐ | ☐ |
| Psychologist / Psychiatrist | ☐ | ☐ | ☐ | ☐ | ☐ |
| Surgeon | ☐ | ☐ | ☐ | ☐ | ☐ |
| Other | ☐ | ☐ | ☐ | ☐ | ☐ |

1. Could the cooperation between different health professions be improved?

- Yes
- No
- Uncertain

1. Are you generally satisfied with your career choice as a chiropractor?
   - Yes
   - No
   - Uncertain
2. Do you currently experience sufficient daily professional challenges? (Present state)

- Yes
- No
- Uncertain

1. Does your income appropriately reflect the time and energy that you put into your work?

- Yes
- No
- Uncertain

*Patient characteristics*

1. How many of your patients are from each of the following gender categories? (Total should be approximately 100%)

| Gender | None | 1–25% | 26–50% | 51–75% | 76–100% |
| --- | --- | --- | --- | --- | --- |
| Male | ☐ | ☐ | ☐ | ☐ | ☐ |
| Female | ☐ | ☐ | ☐ | ☐ | ☐ |
| Other | ☐ | ☐ | ☐ | ☐ | ☐ |

1. How many of your patients are from each of the following age categories? (Total should be approximately 100%)

| Age | None | 1-25% | 26-50% | 51-75% | 76-100% |
| --- | --- | --- | --- | --- | --- |
| 5 or younger | ☐ | ☐ | ☐ | ☐ | ☐ |
| 6–17 | ☐ | ☐ | ☐ | ☐ | ☐ |
| 18–30 | ☐ | ☐ | ☐ | ☐ | ☐ |
| 31–50 | ☐ | ☐ | ☐ | ☐ | ☐ |
| 51–64 | ☐ | ☐ | ☐ | ☐ | ☐ |
| 65–74 | ☐ | ☐ | ☐ | ☐ | ☐ |
| 75–84 | ☐ | ☐ | ☐ | ☐ | ☐ |
| >84 | ☐ | ☐ | ☐ | ☐ | ☐ |

1. What percentage of your patients that you saw IN THE PAST MONTH presented with the following symptom duration? (Total should be approximately 100%.) Please look at the last month before the COVID-19 crisis started in Switzerland (or at a typical month).

|  | None | 1–25% | 26–50% | 51–75% | 76–100% |
| --- | --- | --- | --- | --- | --- |
| Symptom-free | ☐ | ☐ | ☐ | ☐ | ☐ |
| 0–4 weeks | ☐ | ☐ | ☐ | ☐ | ☐ |
| 4–8 weeks | ☐ | ☐ | ☐ | ☐ | ☐ |
| 8–12 weeks | ☐ | ☐ | ☐ | ☐ | ☐ |
| More than 12 weeks | ☐ | ☐ | ☐ | ☐ | ☐ |

1. What percentage of your patients in the past year presented with the following chief complaints? (Total should be approximately 100%.) Please look at the year before the COVID-19 crisis started in Switzerland.

| Percentage | None | 1–10% | 11–20% | 21–30% | 31–40% | 41–50% | 51–60% | 61–70% | 71–80% | 81–90% | 91–100% |
| --- | --- | --- | --- | --- | --- | --- | --- | --- | --- | --- | --- |
| Headache without neck pain | ☐ | ☐ | ☐ | ☐ | ☐ | ☐ | ☐ | ☐ | ☐ | ☐ | ☐ |
| Headache with neck pain | ☐ | ☐ | ☐ | ☐ | ☐ | ☐ | ☐ | ☐ | ☐ | ☐ | ☐ |
| TMD (temporomandibular disorder) without neck pain | ☐ | ☐ | ☐ | ☐ | ☐ | ☐ | ☐ | ☐ | ☐ | ☐ | ☐ |
| TMD with neck pain | ☐ | ☐ | ☐ | ☐ | ☐ | ☐ | ☐ | ☐ | ☐ | ☐ | ☐ |
| Facial pain | ☐ | ☐ | ☐ | ☐ | ☐ | ☐ | ☐ | ☐ | ☐ | ☐ | ☐ |
| Neck pain/injury without arm pain | ☐ | ☐ | ☐ | ☐ | ☐ | ☐ | ☐ | ☐ | ☐ | ☐ | ☐ |
| Neck pain/injury with arm pain | ☐ | ☐ | ☐ | ☐ | ☐ | ☐ | ☐ | ☐ | ☐ | ☐ | ☐ |
| Midback pain/injury | ☐ | ☐ | ☐ | ☐ | ☐ | ☐ | ☐ | ☐ | ☐ | ☐ | ☐ |
| Lower back, pelvis pain/injury without leg pain | ☐ | ☐ | ☐ | ☐ | ☐ | ☐ | ☐ | ☐ | ☐ | ☐ | ☐ |
| Lower back, pelvis pain/injury with leg pain | ☐ | ☐ | ☐ | ☐ | ☐ | ☐ | ☐ | ☐ | ☐ | ☐ | ☐ |
| Upper extremity pain/injury | ☐ | ☐ | ☐ | ☐ | ☐ | ☐ | ☐ | ☐ | ☐ | ☐ | ☐ |
| Lower extremity pain/injury | ☐ | ☐ | ☐ | ☐ | ☐ | ☐ | ☐ | ☐ | ☐ | ☐ | ☐ |
| Chest pain/injury | ☐ | ☐ | ☐ | ☐ | ☐ | ☐ | ☐ | ☐ | ☐ | ☐ | ☐ |
| Abdominal pain/injury | ☐ | ☐ | ☐ | ☐ | ☐ | ☐ | ☐ | ☐ | ☐ | ☐ | ☐ |
| Wellness/preventive care | ☐ | ☐ | ☐ | ☐ | ☐ | ☐ | ☐ | ☐ | ☐ | ☐ | ☐ |
| Other nonmusculoskeletal condition | ☐ | ☐ | ☐ | ☐ | ☐ | ☐ | ☐ | ☐ | ☐ | ☐ | ☐ |
