# Supplementary material for: A descriptive evaluation of a job analysis survey in the chiropractic profession in Switzerland – an update after more than 10 years
Source: Chiropr Man Therap. 2024 Jun 24;32:25. doi: 10.1186/s12998-024-00544-1 (PMC11197174; doi:10.1186/s12998-024-00544-1)
Supplement: Supplementary file 2 — Additional file 2: Supplementary results [file 12998_2024_544_MOESM2_ESM.docx]

# Additional file 2: Supplementary results

*Demographics of chiropractors*

S1 How are you involved in education? (Mark all that apply)

| **Involvement in education** | **Yes**  **(% of chiropractors)** |
| --- | --- |
| As a principal | 17 |
| As a teacher for undergraduate students (bachelor and master level) | 8 |
| As a teacher for postgraduate students (e.g., academy) | 12 |
| As a teacher for continuing education (e.g., annual CE convention, multidisciplinary courses) | 4 |
| As an examiner/as a member of the examining commission | 10 |
| As a clinical supervisor | 10 |
| Other | 7 |

S2 In which of the following continuing education opportunities do you participate? (Mark all that apply)

| **Continuing education opportunities** | **None**  **(% of chiropractors)** | **Chiropractic field**  **(% of chiropractors)** | **Multidisciplinary setting**  **(% of chiropractors)** | **Both**  **(% of chiropractors)** |
| --- | --- | --- | --- | --- |
| Reading journals | 12 | 13 | 16 | 60 |
| Attending conferences/seminars | 1 | 22 | 10 | 67 |
| Attending diploma courses | 50 | 19 | 8 | 23 |
| Attending hospital staff CE meetings | 62 | 6 | 20 | 12 |
| Online credit courses | 55 | 24 | 7 | 15 |
| Courses/seminars offered by Swiss Chiropractic Academy | 8 | 68 | 4 | 20 |
| Other | 66 | 9 | 15 | 10 |

S3 Institution that conferred your chiropractic degree (alphabetic order)

| **Chiropractic college/university of graduation** | **%**  **of chiropractors** |
| --- | --- |
| AECC University College/Anglo-European College of Chiropractic | 5 |
| Canadian Memorial Chiropractic College | 19 |
| Cleveland Chiropractic College | 0 |
| Durban University of Technology | 0 |
| Institut France-Européen de Chiropratique, Ivry-Sur-Seine | 4 |
| Institut France-Européen de Chiropratique, Toulouse | 1 |
| Logan Chiropractic College | 2 |
| Los Angeles College of Chiropractic/Southern California University of Health Sciences | 8 |
| Macquarie University | 0 |
| Murdoch University | 0 |
| National College of Chiropractic/National University of Health Sciences | 5 |
| New York Chiropractic College | 2 |
| Northern Health Sciences University/Northwestern College of Chiropractic | 7 |
| Other | 1 |
| Palmer College of Chiropractic | 12 |
| Palmer College of Chiropractic West | 10 |
| Texas Chiropractic College | 1 |
| Université du Québec à Trois-Rivières | 0 |
| University of Johannesburg | 0 |
| University of Southern Denmark | 0 |
| University of Western States, Chiropractic College | 14 |
| University of Zürich | 10 |
| Welsh Institute of Chiropractic, University of Glamorgan | 0 |

*Mode of operation*

S4 At what time do you reassess your patients for whom you are providing ongoing care

| **Time interval of reassessment** | **% of chiropractors** |
| --- | --- |
| After 1 visit | 21 |
| After 2-3 visits | 23 |
| After 4-5 visits | 35 |
| After 6-7 visits | 14 |
| After 8-9 visits | 3 |
| After 10 or more visits | 3 |
| I do not reassess | 1 |

S5 Please approximate the percentage of your new patients received from the following sources. (Total should be approximately 100%)

| **Source of new patients** | **Mean % of patients** |
| --- | --- |
| ChiroSuisse website | 3 |
| Your own personal website | 15 |
| Patient contact with ChiroSuisse | 2 |
| Sign/location of your practice | 12 |
| Word of mouth/patient referrals | 51 |
| Chiropractic patients association | 3 |
| Referral from medical practitioner | 33 |
| Referral from other chiropractor | 3 |
| Referral from other health care practitioner | 11 |
| Other | 3 |

S6 Percentage of referrals to chiropractic care and of referrals to other health professionals by the chiropractors during the past year (alphabetic order)

|  | **Referrals by other health professionals to chiropractic care (% of chiropractors)**  *("How frequently have the following health care professionals referrals to you during the past year?")* | | | | | **Referrals by chiropractors to other health professionals (% of chiropractors)**  *("How frequently have you made referrals to the following health professionals during the past year?")* | | | | |
| --- | --- | --- | --- | --- | --- | --- | --- | --- | --- | --- |
| **Practitioner** | **Never** | **Rarely**  **(<1/mon)** | **Sometimes**  **(1–3/mon)** | **Often**  **(1–2/wk)** | **Routinely**  **(>2/wk)** | **Never** | **Rarely**  **(<1/mon)** | **Sometimes**  **(1–3/mon)** | **Often**  **(1–2/wk)** | **Routinely**  **(>2/wk)** |
| Acupuncturist | 56 | 39 | 5 | 0 | 0 | 38 | 47 | 14 | 1 | 0 |
| Chiropractor, other | 18 | 67 | 14 | 1 | 0 | 15 | 63 | 19 | 3 | 0.4 |
| Dentist | 39 | 52 | 9 | 0 | 0 | 25 | 61 | 13 | 0.4 | 0 |
| Family practitioner. | 0 | 4 | 24 | 33 | 38 | 0.8 | 12 | 52 | 28 | 7 |
| Gynecologist | 26 | 35 | 22 | 14 | 2 | 32 | 51 | 16 | 1 | 0.4 |
| Internist | 15 | 28 | 33 | 20 | 5 | 22 | 34 | 32 | 9 | 2 |
| Massage therapist | 11 | 40 | 39 | 8 | 2 | 11 | 34 | 39 | 13 | 4 |
| Neurologist | 38 | 40 | 18 | 4 | 0 | 4 | 44 | 42 | 8 | 2 |
| Neurosurgeon | 24 | 36 | 29 | 10 | 1 | 8 | 41 | 43 | 8 | 1 |
| Nutritionist | 76 | 21 | 3 | 0 | 0 | 51 | 44 | 5 | 0.4 | 0 |
| Orthopedic surgeon | 17% | 40 | 25 | 13 | 4 | 3 | 37 | 47 | 10 | 2 |
| Orthopedic technician | 68 | 26 | 5 | 1 | 0 | 36 | 43 | 19 | 2 | 0 |
| Pediatrician | 25 | 47 | 21 | 5 | 2 | 32 | 54 | 12 | 3 | 0 |
| Physiatrist / Rheumatologist | 23 | 40 | 29 | 7 | 1 | 8 | 47 | 40 | 5 | 0.4 |
| Physical therapist | 9 | 40 | 39 | 10 | 1 | 3 | 20 | 41 | 26 | 10 |
| Psychologist / Psychiatrist | 53 | 39 | 7 | 1 | 0 | 32 | 52 | 15 | 0.8 | 0 |
| Surgeon | 49 | 40 | 10 | 1 | 0 | 35 | 46 | 18 | 1 | 0 |
| Other | 90 | 6 | 2 | 1 | 0 | 89 | 8 | 3 | 0.8 | 0.4 |

mon=month; wk=week

*Patient characteristics*

S7: Demographics of patients treated by responding chiropractors in Switzerland

|  | **None** | **1-25% of patients** | **26-50% of patients** | **51-75% of patients** | **76-100% of patients** |
| --- | --- | --- | --- | --- | --- |
| **Gender** (% of chiropractors)  *("How many of your patients are from each of the following gender categories? (Total should be approximately 100%")* | | | | | |
| male | 0.4 | 5.8 | 91.3 | 2.5 | 0.0 |
| female | 0.4 | 0.0 | 39.6 | 58.3 | 1.7 |
| other | 85.9 | 13.7 | 0.4 | 0.0 | 0.0 |
| **Age** (% of chiropractors) | | | | | |
| *("How many of your patients are from each of the following age categories? (Total should be approximately 100%)"* | | | | | |
| ≤5y | 34.9 | 63.9 | 0.4 | 0.4 | 0.0 |
| 6-17y | 2.9 | 95.4 | 1.2 | 0.0 | 0.0 |
| 18-30y | 0.4 | 74.7 | 23.2 | 1.2 | 0.0 |
| 31-50y | 0.8 | 22.0 | 66.8 | 9.5 | 0.4 |
| 51-64y | 0.4 | 31.1 | 61.4 | 5.8 | 0.8 |
| 65-74y | 0.4 | 65.6 | 29.0 | 4.1 | 0.4 |
| 75-84y | 4.1 | 92.9 | 2.5 | 0.0 | 0.0 |
| ≥85y | 12.4 | 87.1 | 0.0 | 0.0 | 0.0 |

y=years

S8: Symptom duration of patients treated by responding chiropractors in Switzerland

|  | **None** | **1-25% of patients** | **26-50% of patients** | **51-75% of patients** | **76-100% of patients** |
| --- | --- | --- | --- | --- | --- |
| **Duration of symptoms** (% of chiropractors) | | | | | |
| Symptom-free | 53.1 | 45.2 | 0.0 | 0.8 | 0.8 |
| 0-4 weeks | 0.4 | 34.0 | 40.2 | 23.2 | 2.1 |
| 4-8 weeks | 1.2 | 51.0 | 42.7 | 5.0 | 0.0 |
| 8-12 weeks | 2.1 | 71.0 | 24.1 | 2.9 | 0.0 |
| >12 weeks | 3.7 | 72.2 | 17.8 | 4.6 | 1.7 |

S9: Location of the main complaint of patients treated by responding chiropractors in Switzerland

| **Main complaint**  (% of chiropractors) | **Percentage of patients** | | | | | | | | | | |
| --- | --- | --- | --- | --- | --- | --- | --- | --- | --- | --- | --- |
|  | **None** | **1–10%** | **11–20%** | **21–30%** | **31–40%** | **41–50%** | **51–60%** | **61–70%** | **71–80%** | **81–90%** | **91–100%** |
| LBP/pelvis pain without leg pain | 0.0 | 2.9 | 29.0 | 31.1 | 15.8 | 10.0 | 7.1 | 2.1 | 1.2 | 0.8 | 0.0 |
| LBP/pelvis pain with leg pain | 0.0 | 13.7 | 37.8 | 24.5 | 11.6 | 6.2 | 3.3 | 1.2 | 0.8 | 0.8 | 0.0 |
| Neck pain without arm pain | 0.4 | 22.4 | 36.5 | 25.3 | 7.5 | 4.1 | 2.5 | 0.4 | 0.4 | 0.4 | 0.0 |
| Neck pain  with arm pain | 0.4 | 39.4 | 40.7 | 10.4 | 4.6 | 2.9 | 0.8 | 0.4 | 0.0 | 0.4 | 0.0 |
| Headache with neck pain | 0.0 | 23.2 | 47.3 | 20.3 | 4.1 | 1.7 | 1.2 | 0.4 | 1.7 | 0.0 | 0.0 |
| Midback pain | 0.0 | 26.1 | 42.7 | 19.1 | 7.5 | 1.7 | 2.1 | 0.0 | 0.8 | 0.0 | 0.0 |

LBP=lower back pain
